# Chronic exposure to insecticides impairs honeybee optomotor behaviour

**DOI:** 10.3389/finsc.2022.936826

**Published:** 2022-08-17

**Authors:** Rachel H. Parkinson, Caroline Fecher, John R. Gray

**Affiliations:** ^1^ Grass Laboratory, Marine Biological Laboratory, Woods Hole, MA, United States; ^2^ Department of Zoology, University of Oxford, Oxford, United Kingdom; ^3^ Department of Biology, University of Saskatchewan, Saskatoon, SK, Canada; ^4^ Institute of Neuronal Cell Biology, Technical University of Munich, Munich, Germany

**Keywords:** imidacloprid, sulfoxaflor, visually guided behavior, optomotor behavior, honeybee

## Abstract

Honeybees use wide-field visual motion information to calculate the distance they have flown from the hive, and this information is communicated to conspecifics during the waggle dance. Seed treatment insecticides, including neonicotinoids and novel insecticides like sulfoxaflor, display detrimental effects on wild and managed bees, even when present at sublethal quantities. These effects include deficits in flight navigation and homing ability, and decreased survival of exposed worker bees. Neonicotinoid insecticides disrupt visual motion detection in the locust, resulting in impaired escape behaviors, but it had not previously been shown whether seed treatment insecticides disrupt wide-field motion detection in the honeybee. Here, we show that sublethal exposure to two commonly used insecticides, imidacloprid (a neonicotinoid) and sulfoxaflor, results in impaired optomotor behavior in the honeybee. This behavioral effect correlates with altered stress and detoxification gene expression in the brain. Exposure to sulfoxaflor led to sparse increases in neuronal apoptosis, localized primarily in the optic lobes, however there was no effect of imidacloprid. We propose that exposure to cholinergic insecticides disrupts the honeybee’s ability to accurately encode wide-field visual motion, resulting in impaired optomotor behaviors. These findings provide a novel explanation for previously described effects of neonicotinoid insecticides on navigation and link these effects to sulfoxaflor for which there is a gap in scientific knowledge.

## Introduction

Prophylactic use of seed treatment insecticides is prevalent, despite growing evidence of negative effects on pollinators (1) and birds (2), and putatively negligible benefits to farmers (3). To overcome insecticide resistance, novel insecticides are continually being developed and implemented in agriculture including sulfoxaflor (a sulfoximine) that is used commercially despite known detrimental effects on bumblebee reproduction (4). Sulfoxaflor and neonicotinoids like imidacloprid act on the same target, the nicotinic acetylcholine receptor (nAChR), but they are thought to be detoxified through different pathways and differ in affinity to nAChR subunits (5, 6). Sulfoxaflor is present in seed treatment mixtures that also include neonicotinoid insecticides and fungicides (e.g., VISIVIO™, Syngenta Seedcare) but no studies have examined the potential for synergy of the toxic effects of these compounds in pollinators.

Neonicotinoid insecticides have been linked with myriad behavioural abnormalities in managed and wild bees, including impaired navigation and foraging (7–9). While these effects are broadly associated with putative deficits in learning and memory, the possibility of neonicotinoids or sulfoximines directly affecting the neural circuitry governing flight navigation in honeybees has not previously been explored. As honeybees are habitually exposed to these insecticides in the field, it is crucial to understand the range of sublethal effects to effectively regulate insecticide use.

In addition to landmark cues, honeybees rely on two primary modes of visual information for navigation: the orientation of polarized sunlight, and optic flow that results from self-motion during flight. While polarized light from the sun is used as a compass (10), the information provided from optic flow (wide-field visual motion) allows bees to maintain a steady flight heading, altitude, and speed, and importantly it functions as a visual odometer with the integration of image velocity over time (11). Bees use wide-field visual motion cues for path integration, which is vital for travelling between the hive and food sources (12). An established technique for monitoring wide field visual motion detection in insects is the optomotor response (13, 14). In response to rotating, vertically oriented black and white sine wave gratings, insects will turn in the direction of movement of the visual scene [for review, see (15)]. The optomotor response is a critical, innate behavior used by insects to regain a stable orientation if accidentally deviated off course during flight.

Here, we showed that sublethal exposure to the cholinergic insecticides imidacloprid, sulfoxaflor or a mixture of these compounds impairs the optomotor behavior in the honeybee. To begin to understand the neural basis of these changes we explored molecular markers of neuronal apoptosis and gene expression. We show that, while these compounds affect detoxification- and stress-related gene expression, the resulting apoptosis in the honeybee brain is minimal and displays high variability between bees. We predict that the insecticides affect optic flow-sensitive neural pathways in honeybees, leading to the impairment of a behavior critical for flight and navigation.

## Methods

### Bees and insecticide exposures

Forager bees (*Apis mellifera*) were collected as they returned to the entrances of four outdoor hives located at Falmouth Academy (Falmouth, MA) between July and August, 2019. To reduce the inclusion of bees affected by age or illness, bees were discarded if they displayed low activity during the first hour after collection or appeared otherwise damaged. There were no nearby fields treated with insecticides, however we do not know whether the bees were exposed to agrochemicals prior to collection. All bees in the study experienced the same field conditions. Bees were housed at the Marine Biological Laboratory (Woods Hole, MA) for 5 days in groups of 2-3 bees in 5 cm^3^ acrylic boxes in a ventilated incubator at 32°C and 55% humidity, which is within the range of thermoregulation of honeybee hives (16). A 1.5 M sucrose solution was provided *ad libitum* to control bees (CTL), and treatment group bees were provided with the 1.5 M sucrose solution containing 50 ppb (195 nM) imidacloprid (IMD, Toronto Research Chemicals, Toronto, Canada), 50 ppb (180 nM) sulfoxaflor (SFX, Toronto Research Chemicals, Toronto, Canada), or a mixture of 25 ppb (97.5 nM) imidacloprid and 25 ppb (90 nM) sulfoxaflor (MIX). Bees from the four hives were allocated equally amongst treatment groups (n=136 bees per hive). Exposure concentrations were selected to reflect field concentrations of sulfoxaflor in nectar [50-900 ppb (17)], although field concentrations of imidacloprid are typically lower [0.5-15 ppb (18, 19)].

### Optomotor assay

**Figure 1:**
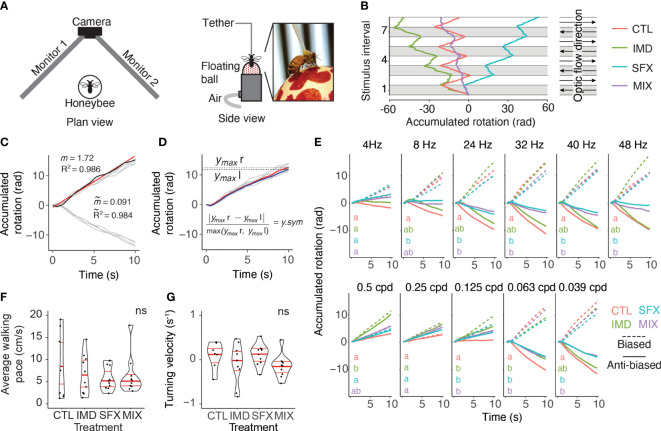
Wide-field visual motion stimulates optomotor behaviors in honeybees. **(A)**, Open-loop virtual reality arena for tethered bees walking on an air-supported ball in response to visual stimulation from two computer monitors. **(B)**, Accumulated rotation of single bees in response to a sequence of leftwards (grey) and rightwards (white) sine wave gratings under control (CTL, left panel) treatment or after exposure to imidacloprid (IMD), sulfoxaflor (SFX) or a mixture (MIX, right panel). In this example, the IMD treated bee displays asymmetrical turning behavior with a leftward bias, the SFX bee displays a rightward bias, and the MIX bee displays shallow turning with a leftward bias. **(C)**, Responses of a single animal to 4 leftward and 4 rightward 16 Hz, 0.0625 cpd optic flow stimuli (light grey lines). Paths are reset to 0 rad and 0 s with each change in stimulus direction, such that leftward motion is positive and rightward is negative, and each stimulus interval begins at 0 s. Linear regressions were fit to each individual path (e.g., red linear regression of dark blue path, with slope, ˜m, and R^2^). Response tortuosity ( *˜R* ^2^) and the absolute turning velocity ( ˜m) are the average R^2^ values and slopes across all 8 linear regressions. **(D)**, Rightward responses were inverted for comparison with leftward responses (same data as in C, light grey lines are individual paths). Maximum accumulated rotation (*ymax*) was averaged across stimulus presentations for a single animal. The mean responses across the 4 rightward (red line, *ymax* r) and leftward (blue line, *ymax* l) were also compared to quantify rotation symmetry (*y.sym*). **(E)**, Average biased and anti-biased responses across all bees per treatment to visual stimuli of varying temporal (top row, constant spatial frequency 0.0625 cpd) and spatial frequencies (bottom row, temporal frequency = 16 Hz). Biased (dashed lines) responses with positive slopes and anti-biased responses (solid lines) with negative slopes match the stimulus direction. There was a significant interaction between stimulus and treatment for the slopes of the accumulated rotation (i.e., turning velocity) in the anti-biased direction (GLMM: *F*_30,497_=1.68, *p*=0.014, random, random effect of individual: $\chi_{1}^{2}$ =216.7, *p* < .0001). Letters denote significant differences in anti-biased slopes between treatments. Number of animals for each treatment and stimulus are listed in **Table S1**. **(F)**, Bees shown images of stationary vertical sine wave bars (no optic flow) walked spontaneously. There was no significant effect of treatment on average walking pace (Kruskal-Wallis, $\chi_{3}^{2}$ =0.12, *p* = 0.99, n=8-13 bees per treatment). **(G)** Turning velocity was not affected by treatment in spontaneously walking bees shown stationary sine wave stimuli (Kruskal-Wallis, $\chi_{3}^{2}$ = 0.57, *p* = 0.13, n=8-13 bees per treatment).

Using an open-loop, virtual reality arena that allows tethered honeybees to walk on an air-supported ball, we tested the effects of chronic exposure to the neonicotinoid imidacloprid (IMD), novel insecticide sulfoxaflor (SFX) and a mixture of the two insecticides (MIX), versus a sucrose control on the optomotor response. The optomotor arena consisted of the air-supported ball, a USB camera module (ELP) positioned 15 cm from the front of the ball, and two computer monitors (CUK Bionic B25GM, 24.5”, 240 Hz refresh rate) oriented at a right angle, such that the bee was facing the apex of the two monitors, 15 cm away from each screen (**Figure 1A**). The refresh rate of the monitors we used (240 Hz) is above the frequency used in other studies exploring wide-field motion detection in honeybees, e.g., 198 Hz (20)). Computer monitors displayed vertically-oriented translating black sine wave bars, creating the illusion of rotational optic flow from the perspective of the bee. Wide-field visual motion elicited the optomotor response in tethered walking bees. The resulting rotation of the air-supported ball was video recorded and the fictive walking path was extrapolated using FicTrac (21) (**Figure 1B**; **Video S1**).

We temperature-anesthetized the bees in a refrigerator at 4°C until unresponsive (10-15 min). Once anesthetized, the dorsal side of the thorax was gently abraded with a blade to remove hairs, and a 1 ml pipette tip was affixed to the dorsal surface of the thorax using UV light-cured dental glue (Prevest DenPro). Bees were then offered a drop of 1.5 M sucrose solution and allowed to recover in an incubator at 32°C for 30 min. After recovery, bees were positioned with a micromanipulator (World Precision Instruments) over the air supported ball in the optomotor arena. A terrarium lamp was positioned to warm the bee until it began to walk unprompted. Bees were excluded from the experiment if they did not start walking within 15 min. This accounted for approximately 15% of the population, irrespective of treatment. In total, n=22 CTL, n=25 IMD, n=28 SFX, and n=25 MIX bees were included in the behavioural assay. All bees were tested with the 16 Hz, 0.0625 cpd stimulus. A subset of these bees were also tested with a range of spatiotemporal frequencies (n=9-16 per stimulus, **Table S1**).

### Visual stimuli

Visual stimuli were programmed in MATLAB R2019b (MathWorks, Natick, MA) by adapting code from the Psychtoolbox-3 toolbox (22). Translating vertical black and white sinusoidal bars were displayed in 10 s intervals, first leftwards on both screens then instantly changing direction to rightwards. This sequence was repeated 4 times, summing to a total of 80 s per stimulus. Twelve visual stimuli were tested, by varying either the spatial or temporal frequency (**Table S1**), and were presented in a pseudo-random order with a 3 min inter-stimulus interval. Stimuli covered spatial frequencies of 0.5 to 0.0039 cycles per degree (cpd), where a smaller value represents wider gratings, which were displayed at a constant temporal frequency of 16 Hz. In addition, temporal frequencies from 4 to 48 Hz were tested at a constant spatial frequency of 0.0625 cpd. The spatial frequencies were selected based on Ibbotson et al. (20), which demonstrated that descending neurons respond to spatial frequencies between 0.028 and 0.071 cpd and velocities at and above 200 deg/s^-1^. We included stimuli outside this range (i.e., with very small spatial frequencies and low velocities), which was necessary to quantify how bees perform when the stimulus is either not visually resolvable or does not elicit the optomotor response for comparison of pesticide treated bees with the control. A subset of animals were also tested with stationary black bars to measure differences between treatments in walking behavior in the absence of optic flow.

### Fictrac video analysis

Optomotor behavior tracking was performed online using FicTrac (21). A program was custom-written in Matlab to align the FicTrac data to the visual stimuli and extract the accumulated rotation and total movement across all axes (**Figure 1B**). The accumulated rotation and time were reset to 0 at the initiation of each stimulus and each stimulus direction switch (e.g., leftward to rightward) such that the output for leftward turns was positive, while rightward turns produced negative values and each stimulus interval commenced at time=0 (**Figure 1C**).

We fit linear regressions to the 8 normalized response replicates (4 replicates per direction) for a given animal-stimulus pair (**Figure 1C**). The slopes of the linear regressions for each of the four leftward and rightward stimuli (i.e., turning velocity) were averaged to assess the symmetry of the behavioural responses. Absolute turning velocities of 0 represented perfectly symmetrical leftward versus rightward responses, while slopes of increasing magnitude signified greater response asymmetry. The R^2^ values for each linear regression were also averaged to produce a measure of path tortuosity. Responses that switched direction slowly or those that wavered from left to right produced lower R^2^ values than responses that quickly switched direction to follow the visual stimulus and maintained a consistent yaw for the duration of the stimulus. Movement distance (across all rotational axes) and diameter of the air supported ball were used to calculate the walking pace and this was averaged across the 4 leftward and 4 rightward stimulus presentations (80 s of walking behavior).

To compare the magnitude of leftward and rightward optomotor responses, we changed the sign of the rightward responses so that both directions of responses were on the same scale (i.e., both positive slopes, **Figure 1D**). We calculated the response magnitude as the maximum accumulated rotation (*ymax*) averaged over stimulus presentations in both directions. Maximum accumulated rotation was also compared between the leftward and rightward stimuli to determine if the responses were asymmetrical. Average *ymax* values were calculated separately for leftward and rightward responses, and rotation asymmetry (*y.sym*) was obtained with:


(1) $$\frac{\left|ymax\;r-ymax\;\text{l}\right|}{max\left(ymax\;r,ymax\;\text{l}\right)}=\text{y}.\text{sym},$$

where *ymax* r is rightward and *ymax* l is leftward maximum accumulated rotation. Values of *y.sym* close to zero represented symmetrical responses, while highly asymmetrical responses had *y.sym* values closer to 1. Responses were labeled “biased” in the direction that elicited the larger response and “anti-biased” in the other direction.

### Relative quantification of gene expression

A separate set of bees were chronically exposed to IMD, SFX, MIX, or sucrose (CTL) for 5 days, as described above (n=25 bees per treatment were allocated randomly from 4 colonies). Freshly dissected honeybee brains were snap frozen on dry ice and stored at -80°C until further use (≤2 weeks). The material from 5 bee brains was pooled to one sample and 5 samples were generated per treatment group. Total RNA was isolated from each sample using the Maxwell^®^ RSC miRNA Tissue Kit (Promega) and the Maxwell^®^ RSC 48 Instrument (Promega) according to the manufacturer’s instructions. RNA concentration was measured using the QuantiFluor^®^ RNA System (Promega), and total RNA was quantified by Quantus Fluorometer (Promega) following manufacturer protocol, yielding between 120 - 210 ng/μl RNA and a total of 7.2 - 12.6 μl.

Neonicotinoid insecticides have previously been shown to cause neural inactivation and cell death in the honeybee brain (23, 24). One mechanism of action hinges on oxidative damage resulting from reactive oxygen species generated during the detoxification of the insecticide (25). To assess whether IMD, SFX, or MIX affect gene expression in the honeybee central nervous system, we performed one-step reverse transcriptase (RT)-qPCR on the brains of honeybees exposed to the same treatments used for the behavioral assay. We examined the relative gene expression (versus expression in untreated bees) of two cytochrome P450 enzymes (CYP9Q2, CYP9Q3) involved in the detoxification of insecticides, as well as two enzymes involved in scavenging reactive oxygen species, superoxide dismutase 1 (SOD1) and catalase (CAT). Genes of interest were superoxide dismutase 1 (SOD1), catalase (CAT), cytochrome P450 enzymes (CYP9Q2 and CYP9Q3), and the reference gene GAPDH. Forward and reverse primer sequences and sequence accession numbers are described in **Table S2**. Primers were either designed using NCBI Primer BLAST (NCBI) with 100% specificity for the genes of interest or taken from previous studies (26, 27). RT-qPCR was performed with a 1-step method combining reverse transcription and amplification in a single tube using the GoTaq 1-Step RT-qPCR System (Promega). A 20 μl final volume contained 10 μl GoTaq qPCR Master Mix, 0.4 μl GoScript 1-step RT Master Mix, 0.5 μl of 10 μM forward and reverse primer mix (Genewiz, New Jersey), 7.1 μl Nuclease Free Water, 0.8 μl MgCl_2_ and the RNA sample. Samples were loaded into a QuantStudio 5 (ThermoFisher Scientific) and run with the following cycling conditions: 45°C for 15 minutes, 95°C for 10 minutes, 40 cycles of 95°C for 15 seconds and 60°C for 30 seconds (data collection step), and finally 72°C for 60 seconds followed by the melt curve. RT-qPCR amplification was assessed with the normalized fluorescent signal (Delta Rn) from each sample (**Figure S1A**). We used 1 – 0.0001x dilution series of each RNA sample and the standard curve was used to demonstrate that the presence of PCR inhibitors was unlikely (**Figure S1B**). A reaction without RT enzyme was used as reaction control for assessing the absence of DNA in all samples: no amplification could be detected in these samples.

We validated qPCR specificity using the melt curve analysis (**Figure S1C**). Samples resulting in qPCR efficiency below 90% were omitted from the analysis (3 of 20 samples). Data analysis was performed using QuantStudio5 (ThermoFisher Scientific) using the Cq threshold method (28, 29). RNA amplification of genes of interest were normalized to the amplification of GAPDH for each sample (delta Ct), and then compared to the mean amplification of the control samples (delta delta Ct).

### TUNEL assay

An additional cohort of honeybees (n=4 per group, one bee from each hive) were treated with IMD, SFX or sucrose (CTL) for 5 days as described above. For tissue processing, cold-anesthetized honeybees were decapitated, the brain was dissected under cold PBS, pH 7.4 (as previously described by (30)) and immersion fixed in 4% PFA/PBS, pH 7.4 at 4°C overnight. The tissue was washed in PBS for 12 hours at 4°C and incubated in 30% sucrose/PBS at 4°C overnight. Prior to sectioning, tissue was immersed in Tissue-Tek O.C.T. compound (Sakura) and frozen on dry ice. Brains were sectioned at 12 μm using a cryostat, collected on SuperFrost Plus adhesion slides (Thermo Scientific), and stored at -20°C until further use. Two sections per brain (120 μm cutting distance) were mounted on each slide.

For TUNEL assay, tissue was defrosted and washed 5 times with PBS, pH 7.4. Sections were permeabilized in 0.1% Triton X-100, 0.1 M sodium citrate pH 6.0 at RT for 30 minutes. After 4 washes with PBS, sections were treated with TUNEL reagent (*In situ* cell death detection kit, fluorescein; Roche) in a dark, humidified 37°C chamber for 3 hours according to the manufacturer’s instructions. As negative control, no enzyme solution was applied during this step (**Figure S2**). As positive control, sections were treated with DNase I (1.5 U/μl) in 10 mM Tris-HCl, pH 7.4, 10 mM MgCl_2_, 1 mg/ml BSA at 37°C for 60 minutes prior to TUNEL development (**Figure S2**). All sections were washed 3 times with PBS and incubated with Hoechst 33342 (1:1000) in PBS for 10 minutes. After 2 washes in PBS, sections were mounted with Fluoromount aqueous mounting medium (Sigma-Aldrich).

Images were acquired using an upright FV1000 confocal microscopy system (Olympus) equipped with a 20x/N.A. 0.85 oil immersion objective and using the laser lines 405 nm for Hoechst and 488 nm for TUNEL with standard filter sets. Image analysis was further performed using the open source software ImageJ/Fiji (31). We selected areas of interest (MB, La, Lo, Me) in the honeybee brain and quantified TUNEL+ cells only for areas rich in nuclei (Hoechst staining); hence, excluding the neuropil (**Figure 5A**). Under blinded conditions, the cells within this area were defined for 2-4 sections per bee. Sections with poor Hoechst staining intensity were excluded from the analysis. We did not observe areas with obviously high apoptosis in brain areas not quantified in this analysis.

### Consensus clustering and other statistical analyses

We performed all statistical analyses in R version 4.0.3 (32). Optomotor response types were clustered using a k-means algorithm to group the five behavioral response features (maximum accumulated rotation, tortuosity, walking pace, absolute turning velocity, and rotation asymmetry) across treatments and visual stimuli. In total there were 612 responses from 115 bees in the 4 treatments. Prior to clustering, response features were z-scored. Optimal cluster count was predicted using Monte Carlo reference-based consensus clustering [M3C (33)], an unsupervised learning technique that aggregates the results of multiple clustering runs. Consensus clustering was confirmed and implemented using ConsensusClusterPlus (34)). For each clustering iteration, we included 90% of the total responses and 80% of the behavioral response features (4 of 5). The clustering algorithm was repeated for 2000 iterations, and the output from each clustering run was compared to define a consensus matrix. The proportion of behavioral responses assigned to each cluster was defined for each treatment and visual stimulus. The behavioral responses within each cluster were then compared to provide a quantitative illustration of the different response types across the visual stimuli and treatments.

Data were tested for normality using the Shapiro-Wilk normality test and equal variance using Levene’s test from the car package (35). In the lmerTest (36) and nlme (37) packages we used generalized linear mixed models (GLMM) to compare features of behavioral responses across treatments and stimuli, with animal as a random effect to account for repeated measures (response∼treatment*stimulus+(1|bee)). Models explored whether the interaction between treatments and stimuli were significant. Models with non-significant interaction terms were re-run without the interaction term (response~treatment+stimulus+(1|bee)). Gaussian distributions were used to model the data for accumulated rotation, average walking pace, and absolute turning velocity, while beta regression distributions were used for the rotation asymmetry and tortuosity. The selection of the most parsimonious model for GLMM was carried out assessing the relative importance of each fixed factor with a stepwise subtraction method. This approach with hypothesis testing based on comparing nested models uses the functions anova or lrtest. The latter function was applied for the beta regression with the lmtest package. Cluster assignment was analyzed with GLMM and binomial distributions, by testing the occurrence of Cluster 1 responses across stimuli and treatments (cluster 1~treatment+stimuli+(1|bee), and the distribution of cluster 2 vs cluster 3 for the remaining responses (cluster 2~treatment+stimuli+(1|bee). *Post hoc* multiple comparisons were performed using the emmeans package (38) with the Kenward-Roger degrees-of-freedom method and Tukey p-value adjustments to account for multiple statistical tests. Average consumption, relative gene expression, and spontaneous (no stimulus) behavioural responses were quantified with Kruskal-Wallis rank sum tests with Mann Whitney U test pairwise comparisons with Bonferroni corrections. The TUNEL quantification could not be assessed statistically due to low sample sizes in the SFX treatment. Statistical significance was assessed at *p*<0.05. In figures, (*, <0.05; **, <0.001; ***, <0.0001) statistical significance from *post hoc* multiple comparisons, or letters show significant effects between groups.

## Results

### Exposure to pesticides impairs optomotor responses

Over the 5-day exposure period there were no significant differences in the volume of solutions consumed or survival between the control and pesticide treatment groups (**Figure S3**). Bees consumed on average 46 μl of sugar solution per day, resulting in average doses of 2.3 ng imidacloprid/bee/day (IMD), 2.3 ng sulfoxaflor/bee/day (SFX), or 1.12 ng imidacloprid/bee/day + 1.12 ng sulfoxaflor/bee/day (MIX, **Table 1**).

**Table:** Table 1. Average volume of sugar solution and active ingredient (a.i.) consumed.

Treatment	Avg volume (μl/bee/day)	sd	Dose (ng a.i./day)
CTL	46.2	5.2	none
IMD	46.1	4.9	2.30
SFX	46.0	3.7	2.30
MIX	45.0	4.6	1.12 each

We tested the optomotor responses of honeybees in an open-loop arena using translating vertical sine-wave gratings displayed on two computer monitors to create the illusion of rotational optic flow (**Figure 1A**). Optomotor responses were elicited with a range of spatiotemporal grating frequencies (**Table S1**). The walking paths of honeybees were extracted from video and optomotor responses were quantified using the accumulated rotation of the responses (**Figures 1B**–**D**). Responses to 4 leftward and 4 rightward stimuli were averaged for a single animal, and there was no effect of pesticide treatment on the variability of responses across replicates (**Figure S4**). In control (untreated) bees, optic flow visual stimuli elicited characteristic optomotor behaviors that were tuned to angular velocities >200 deg/s (**Figure 1E**). Stimuli with small spatial frequencies (0.5-0.125 cpd) failed to elicit optomotor responses, as shown previously (15). We exposed separate groups of bees to imidacloprid, sulfoxaflor, or a mixture of these compounds. Stimuli that elicited robust optomotor responses in control bees elicited weaker responses in bees from the pesticide treatment groups. Bees displayed asymmetrical optomotor responses, such that the accumulated rotation was greater for responses to stimuli in one direction (“biased” response) than the other (“anti-biased” response, **Figure 1E**, GLM: *F*_1,1132_ = 190.7, *p*<0.0001). In the anti-biased direction, there were significant differences in the turning velocity between treatments (**Figure 1E**). There were no effects of the insecticides on walking pace or turning velocity in the absence of visual stimulation (**Figures 1F**, **G**). We observed that pesticide-exposed bees were similarly active as control bees while caged, and the incidence of bees that would not walk on the air-supported ball (approximately 15%) was consistent with the control group and other studies (39).

**Figure 2:**
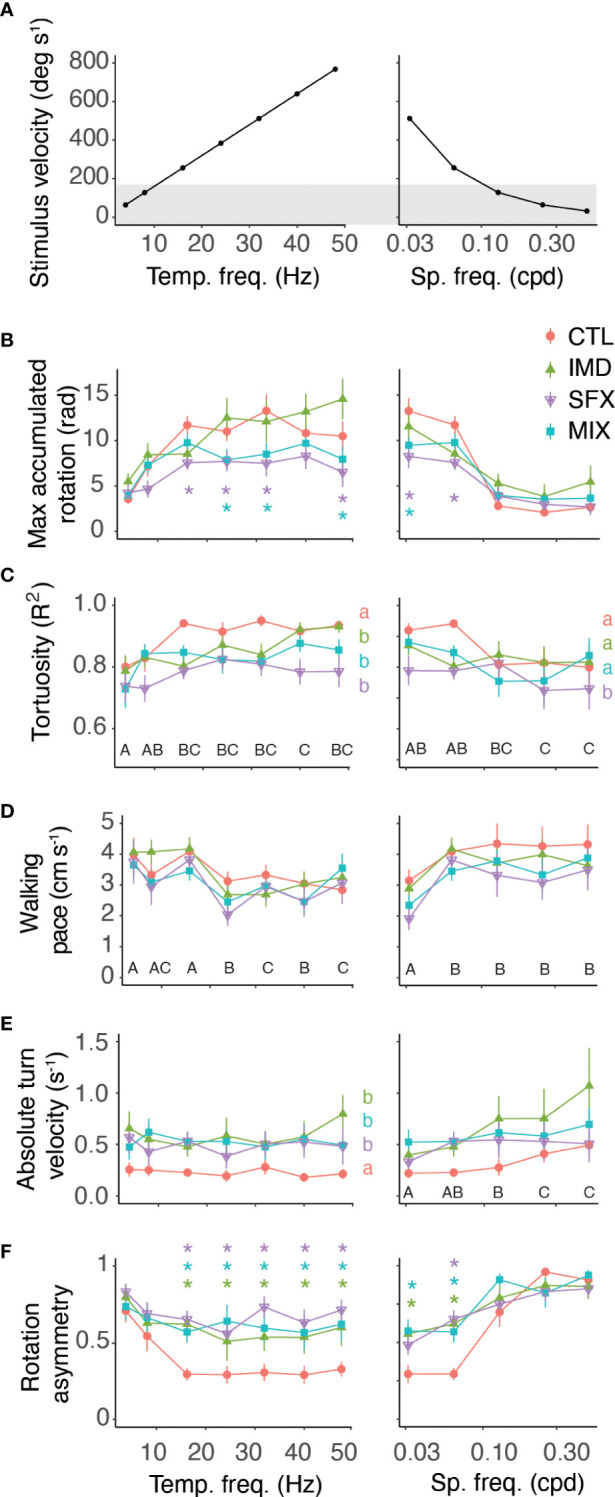
Behavioural response features vary by treatment and optic flow frequencies. **(A)** Stimulus velocity as a function of temporal frequencies (left, constant spatial frequency of 0.0625 cpd) and spatial frequencies (right, constant temporal frequency of 16 Hz). Grey shading represents stimuli that did not elicit robust optomotor responses in control bees. **(B–F)** Behavioural response features with optic flow of varying temporal (left panels) and spatial (right panels) frequencies, showing the mean ± s.e.m. across animals in each treatment group (n=23 to 28 bees per treatment). Results of *post hoc* tests (emmeans) shown on figures with letters denoting significant effects of pesticide treatments (lowercase) versus the control and stimuli (uppercase) when there is no interaction, and colored asterisks showing significant differences for specific stimuli and treatments when there is an interaction. Statistics reported in **Table 2**.

We compared various behavioural response features across the range of spatiotemporal stimulus frequencies and treatments (**Figure 2A**). Bees treated with sulfoxaflor (SFX) or the mixture (MIX) displayed lower accumulated rotation compared to the control (**Figure 2B** and **Table 2**). Path tortuosity was affected by pesticide treatments across stimulus temporal frequencies resulting in a lower R^2^ when fitting the path to a linear regression (**Figure 2C** and **Table 2**). Low R^2^ values resulted from bees that had slow reaction times to switch turning direction in response to the stimulus, and those that displayed meandering walking paths. Walking pace, however, was not affected by pesticide exposure, although it did vary by stimulus spatiotemporal frequency (**Figure 2D** and **Table 2**). Bees walked slower in response to stimuli that elicited robust optomotor responses (i.e. with velocities >200 deg/s^-1^).

**Table 2:** Generalized linear mixed model outputs, including the standard deviation of the random effects for the behavioural response features in **Figure 3**.

	Varying temporal frequency				Varying spatial frequency			
Feature	treat	stim	treat*stim	random	treat	stim	treat*stim	random
Accum. rota- tion			*F*_18,288_ *=* 2.80 *p*=0.001	*sd* = 3.64			*F*_12,211 =_ 2.78 *p*=0.002	*sd = 3.18*
Tortu -osity	*F*_3,84_ = 8.64, *p<*0.0001	*F*_6,326_ = 5.87, *p<*0.001		*sd* = 0.06	*F*_3,98_ *=* 3.57, *p*=0.02	*F*_4,245_ = 4.73, *p*=0.001		*sd* = 0.06
Avg walking pace	*F*_3,95_ = 0.79, *p*=0.50	*F*_6,302_ = 11.89, *p<*0.0001		*sd* = 1.4	*F*_3,98_ *=* 1.03, *p*=0.38	*F*_4,223_ = 12.48, *p*<0.0001		*sd* = 1.6
Abs turn velocity	*F*_3,99_ = 3.06, *p*=0.03	*F*_6,306_ = 0.705, *P*=0.65		*sd* = 0.35	*F*_3,105 =_ 1.89, *p*=0.14	*F*_4,235_ = 5.53, *p*<0.0001		*sd* = 0.41
Rota- tion asym.	*F*_3,94_ = 6.93, *p<*0.0001	*F*_6,313_ = 10.16, *p<*0.0001		*sd* = 0.21			*F*_12,219 =_ 2.71, *p* = 0.002	*sd* = 0.15

We observed that a large proportion of bees treated with the pesticides displayed asymmetrical optomotor responses across the range of stimuli that we tested. Control bees also occasionally displayed asymmetrical responses, but this effect tended to occur primarily for stimuli with very small spatial frequencies. To quantify whether this bias was an artifact of the experimental setup, or a real effect of the insecticide treatments, we averaged the turning velocities of leftward (positive slopes) and rightward (negative slopes) walking paths of responses (˜m, **Figure 1C**). We used the absolute turning velocity so that leftward or rightward biases were accounted for on the same scale. Perfectly symmetric responses to leftward and rightward visual motion resulted in slopes (turning velocities) equal to zero. We found that a turn bias existed to some degree across all treatments, however this bias was significantly greater with pesticide-treated bees (**Figure 2E** and **Table 2**). The bias in the absolute turning velocity does not, however, take into consideration the magnitude of the responses in either direction. To account for this, we compared the difference in the maximum accumulated rotation of responses to leftward and rightward visual motion (*y.sym*, **Figure 2F** and **Table 2**). Control bees displayed a rotation asymmetry of approximately 25% for stimuli that elicited robust responses, while the asymmetry was >60% across all stimuli for bees in the pesticide treatment groups. Interestingly, the turn bias increased significantly in the responses of control bees to stimuli with low velocities.

### Consensus clustering revealed three behavioural response types

Overall, features of optomotor responses across the range of spatiotemporal frequencies showed that control bees displayed weak optomotor responses to stimuli with low temporal frequencies (<16 Hz), and displayed very high levels of response asymmetry (>90%) for stimuli with small spatial frequencies (0.125-0.5 cpd). While the low temporal frequency stimuli may simply not provide sufficient angular velocity to elicit robust responses, the low spatial frequency stimuli are also at the honeybee’s limit of visual resolution (40). Responses of the control bees to these stimuli, thus, represent examples of how bees behave in this paradigm when the visual motion does not stimulate the innate optomotor response. The behavioural response features of pesticide-treated bees displayed reduced variation across the spatiotemporal stimulus frequencies, although these multidimensional effects were difficult to compare between treatments and stimulus types. To more effectively characterize the different response types, we employed a consensus clustering algorithm on the five behavioural response features (maximum accumulated rotation, tortuosity, walking pace, absolute turning velocity and rotation asymmetry), which predicted an optimal cluster count of 3 (**Figure S5**).

**Figure 3:**
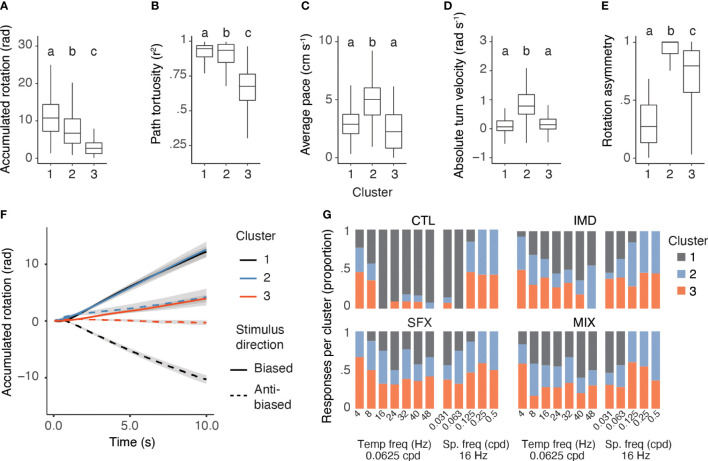
Characterization of response types shows a low rate of successful optomotor behaviors in pesticide treated bees. **(A–E)**, Behavioural response features are significantly different between clusters, irrespective of treatment or stimulus, including the maximum accumulated rotation **(A)**, GLMM: F_2,594_ = 224.85, p<0.0001, random effect of individual: $\chi_{1}^{2}$ = 165.81, p<0.0001), path tortuosity **(B)**, GLMM: F_2,600_ = 372.16, p<0.0001, random effect of individual: $\chi_{1}^{2}$ = 40.25, p<0.0001), average walking pace **(C)**, GLMM: F_2,570_ = 75.69, p<0.0001, random effect of individual: χ^2^_1_ = 290.37, p<0.0001), turn velocity **(D)**, GLMM: F_2,568_ = 47.63, p<0.0001, random effect of individual: $\chi_{1}^{2}$ = 211.54, p<0.0001), and rotation asymmetry **(E)**, GLMM: F_2,606_ = 543.2, p<0.0001, random effect of individual: $\chi_{1}^{2}$ = 63.52, p<0.0001, Cluster 1: n=216 responses, Cluster 2: n=187 responses, and Cluster 3: n=209 responses letters denote significant differences between clusters). **(F)**, Mean responses in each cluster (with confidence intervals). Responses to leftward stimuli = solid, rightward = dotted lines. **(G)**, Proportion of behavioural responses assigned to each cluster across visual stimuli and treatments. Letters denote significant differences between clusters.

We compared the behavioural response features from each cluster irrespective of treatment or stimulus type, and found that the clustering algorithm was successful in distinguishing responses using all parameters (**Figures 3A**–**E**). This was important, as similarity in one parameter (e.g. absolute turning velocity) does not always correspond with similarity in another (e.g. maximum accumulated rotation). The responses were distinct: Cluster 1 was characterized by symmetrical and robust optomotor responses, with biased and anti-biased stimuli eliciting robust turning in the same direction as the visual motion; Cluster 2 responses were faster walking paces and highly asymmetrical. Responses in this cluster included large magnitude turns in one direction that were either uninterrupted or only shallowly corrected with the change in stimulus direction; and Cluster 3 represented responses with low magnitude (i.e., walking forwards rather than turning strongly) and a shallow turn bias (**Figure 3F**). We concluded that Cluster 1 represented robust optomotor responses, while Clusters 2 and 3 contained weak or unsuccessful optomotor responses of two distinct types.

The three response types were found across stimuli and treatments in varying proportions, with the highest proportion of control bees displaying Cluster 1 responses to stimuli that evoked the most robust optomotor responses (e.g., 16-48 Hz and 0.0313-0.0625 cpd, **Figure 3G**). Cluster 2 and 3 responses were represented at higher proportions for stimuli that evoked very weak/no optomotor responses in the control bees (0.125-0.5 cpd, 4-8 Hz), while they were represented for all visual stimuli at high proportions for the pesticide treatment groups (**Figure 3G**). Overall, the proportion of Cluster 1 responses was significantly higher for the control group versus the pesticide treatments (GLMM, treatment: $\chi_{3}^{2}=26.51$ , p<0.0001; stimulus: $\chi_{10}^{2}=70.53$ , p< 0.0001, n=22-28 bees per treatment).

We assessed whether either of the unsuccessful optomotor response types (Clusters 2 and 3) varied between the control and pesticide treatment groups. Both response types were represented for stimuli with very small spatial frequencies (0.25 and 0.5 cpd, 57% Cluster 2, 43% Cluster 3) for control bees. Pesticide-treated bees displayed increased Cluster 2 and 3 responses across stimuli (**Figure 3G**). However, the proportion of Cluster 2 versus Cluster 3 responses did not vary by treatment, despite small differences across stimuli (GLMM, treatment: $\chi_{3}^{2}=0.787$ , p = 0.85; stimulus: $\chi_{10}^{2}=18.39$ , p = 0.049, n = 22-28 bees per treatment).

### Gene expression in the honeybee brain is affected by chronic pesticide exposure

**Figure 4:**
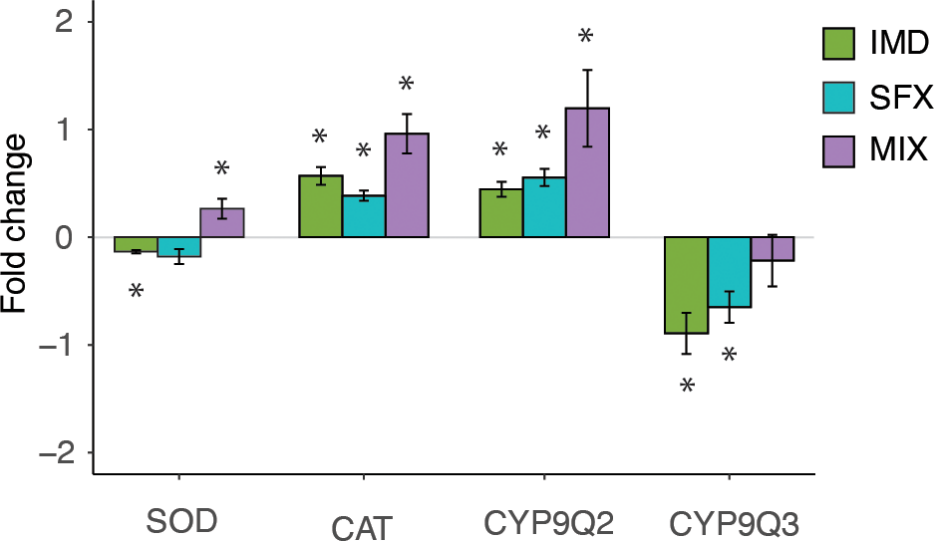
Chronic sublethal pesticide exposure affects gene expression in the bee central nervous system.Fold change of gene expression versus relative control expression levels for imidacloprid (IMD), sulfoxaflor (SFX), and a mixture (MIX). Pesticide treatment affects gene expression of superoxide dismutase (SOD, Kruskal-Wallis, $(\chi_{3}^{2}$ = 13.9, *p*<0.01), catalase (CAT, $\chi_{3}^{2}$ = 14.7, *p*<0.01), and detoxification enzymes CYP 9Q2 $(\chi_{3}^{2}$ =15.7, *p*<0.001), and CYP 9Q3 $(\chi_{3}^{2}$ = 10.3, *p*<0.01), in the honeybee brain. Mann Whitney U test *post hoc* analyses comparing the pesticide treatments to the control (0 fold change) denoted with asterisks (*, p<0.05). Bars show means ± s.e.m. (n=5 per assay).

The optomotor behavior requires accurate processing of visual information in the brain. We hypothesized that the impaired behaviors we observed following insecticide treatment resulted from the influence of the insecticides on the nervous system. To begin to understand the neural mechanism, we measured gene expression in the honeybee brain. Compared to the control condition, IMD resulted in decreased expression of SOD1, while MIX slightly increased expression (**Figure 4**). All pesticide treatments resulted in significantly increased expression of CAT and CYP9Q2, while CYP9Q3 expression was significantly decreased after treatment with IMD or SFX only (**Figure 4**). Interestingly, MIX (25 ppb IMD and 25 ppb SFX) treatment resulted in a greater increase in gene expression than from IMD or SFX separately (50 ppb each) for CAT and CYP9Q2, suggesting an interaction of IMD and SFX in these pathways.

### Sulfoxaflor results in sparse apoptosis in the optic lobes

**Figure 5:**
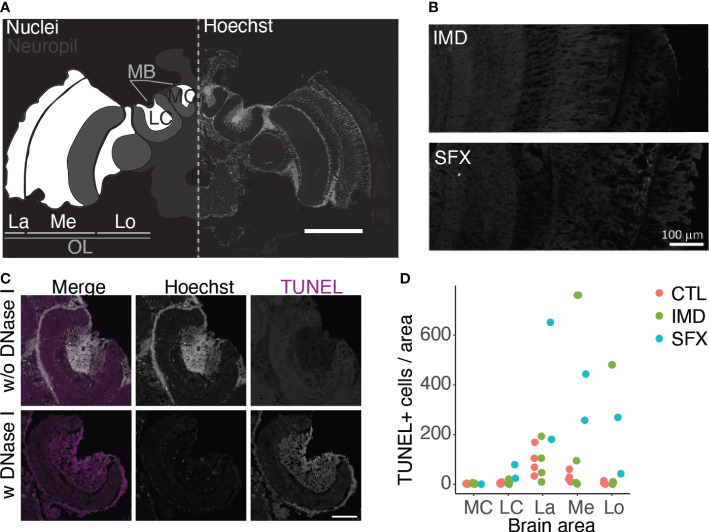
Sulfoxaflor results in sparse increases in apoptosis in the optic lobes. **(A)**, Confocal image of a honeybee brain with Hoechst staining. The areas of interest for TUNEL quantification are highlighted in white on the left side, including the lateral (LC) and medial calyces (MC) of the mushroom bodies (MB), and the lamina (La), medulla (Me) and lobula (Lo) of the optic lobe (OL, scale bar = 500 μm. **(B)**, Images of the optic lobes following treatment with IMD and SFX, with TUNEL staining (scale bar = 100 μm). **(C)**, Positive and negative controls for TUNEL staining in the mushroom bodies (scale bar = 100 μm). **(D)**, Quantification of TUNEL+ cells (normalized to the area factor) in the brain areas of interest. Data points represent mean values across 2 - 4 sections per brain.

Altered expression of stress genes may indicate oxidative damage which can result in apoptosis. We performed a TUNEL assay to visualize apoptosis in the honeybee brain following chronic exposure to IMD or SFX. We quantified apoptotic cells in the optic lobes and mushroom bodies as areas of interest (**Figures 5A–C**). Quantification revealed a trend toward more apoptotic cells in the optic lobes following exposure to SFX (**Figure 5D**). The medial calyx (MC) and lateral calyx (LC) of the mushroom bodies (MB) contained very few apoptotic cells across treatments. We observed apoptotic cells sparsely distributed in the lamina (La), medulla (Me) and lobula (Lo) of the optic lobes, however their number varied by subject (**Figure 5D**).

## Discussion

Myriad insecticides and other agrochemicals are present in the environment at concentrations that are below the lethal dose for pollinators like honeybees. While many studies report toxic effects of cholinergic insecticides at sublethal quantities, there is an incomplete understanding of the relationship between insecticide exposure and visually-guided behavior. We show here, for the first time, that chronic sublethal exposure to imidacloprid, sulfoxaflor, or a mixture of these insecticides results in impaired optomotor behaviors in the honeybee. We hypothesize that these effects result from impairments in the processing of wide-field visual information. Honeybees rely on accurate wide-field motion detection for flight stabilization, and additionally for path integration to return to the hive after foraging bouts (41). Using the optomotor behavior as a proxy for wide-field motion detection (14), we showed that insecticide-exposed bees display poor behavioural responses to rotational optic flow. This was indicated by asymmetrical or shallow turning responses to visual stimuli that elicited strong optomotor responses in control bees. The unsuccessful response types matched those performed by control bees when shown gratings with very small spatial frequencies, suggesting that the pesticide-treated bees may have been unable to perceive the gratings and direction of motion. Other possible explanations for the asymmetrical response type hinge on putative asymmetrical effects of the insecticides in the brain, or effects on head roll stabilization which could impair the magnitude of the response in one direction (42). These putative causes should be explored in a future study. We found that, compared to imidacloprid, sublethal sulfoxaflor exposure resulted in a somewhat larger effect on visual motion-guided behavior. This contrasts effects of these insecticides on the locust, where imidacloprid alone had effects on visual motion detection in the sublethal dose range (e.g., 10 ng/locust (43)), and suggests that bees are more sensitive to the neurotoxic effects of sulfoxaflor.

Visual motion detection depends on precise temporal relationships between spatially organized inputs and is driven by cholinergic neurotransmission in insects (44). One of the most important mechanisms neural circuits employ for dynamic adjustment of sensory input and motor output is gain modulation. Gain modulation filters undesired sensory input and amplifies salient sensory cues with changing environmental demands (45). In the context of visual motion detection, gain control is crucial for maintaining a large dynamic range of visual motion speed and angular size, and is modulated by state and attention (46, 47). Excitatory signalling within insect visual circuits is achieved primarily via nAChRs localized along the synapses of visual motion-sensitive neurons in the optic lobes (44). Gating of information flow in these circuits, therefore, is likely mediated by the kinetics of these receptors, which depend on nAChR subunit composition (48). Exposure to neonicotinoids affects nAChR expression (6, 49–51) which is a homeostatic mechanism to reduce toxicity following agonist exposure. In the locust, imidacloprid alters the response profiles of descending visual motion-sensitive neurons, indicative of a decrease in excitation in the optic lobes (43). Imidacloprid also decreases wide-field motion responses of descending neurons in hoverflies (52). We hypothesize that the effects we observed on the optomotor response result from impaired visual motion processing, an effect which may arise from altered gain modulation in the optic lobes.

We measured gene expression in the honeybee brain to quantify detoxification and homeostatic mechanisms in response to insecticide toxicity. Expression changes of stress and detoxification genes following neonicotinoid or sulfoxaflor exposure in the honeybee has been explored previously, with a high degree of variability dependent on exposure method and duration (49, 53, 54). CYP9Q2 and CYP9Q3 enzymes are known to be involved in insecticide detoxification in honeybees (55), although it had not previously been shown whether these enzymes detoxify sulfoxaflor. In our assay, both imidacloprid and sulfoxaflor (50 ppb exposures) resulted in the upregulation of CYP9Q2 and downregulation of CYP9Q3, suggesting that these insecticides are detoxified through similar pathways in the honeybee. However, the regulation of these pathways likely differs, as the mixture (25 ppb IMD, 25 ppb SFX) failed to induce CYP9Q3 expression, while CYP9Q2 was upregulated. This was also true for catalase, which was upregulated more by the mixture than the individual compounds. Binary mixtures of insecticides have previously been shown to differentially affect gene expression in honeybees (50), and this deserves attention as insecticides are often applied in mixtures (56). We additionally found that superoxide dismutase 1 was downregulated by imidacloprid while it was upregulated by the mixture. Altered regulation of catalase and superoxide dismutase 1 is indicative of oxidative stress, which has been shown to arise from calcium excitotoxicity following neonicotinoid exposure in *Drosophila* (57). Oxidative stress can result in neural apoptosis, which could damage motion detection circuits in the optic lobes. We found that sulfoxaflor resulted in increased apoptosis in the optic lobes, although there was a high degree of variability between animals, and in some cases no effect compared to the control. We conclude that, while apoptosis may enhance the behavioural effects we observed, this is likely not the primary mechanism affecting the optomotor response. Previous studies found increases in apoptosis following exposure to imidacloprid in the honeybee brain, and these contradictory results may in part be explained by differences in exposure periods (e.g., 6-8 days (24, 58).

Another possible explanation for the effect we observed of insecticides on visually guided behavior involves direct toxicity to the motor pathways controlling walking, as neonicotinoids have been shown to affect flight behavior in bumblebees (59), locusts (60) and honeybees (61). However, we did not observe any differences between treatments in spontaneous walking behavior or the average walking pace across the spatial and temporal grating frequencies. This suggests that the motor control of walking was not affected by the insecticides. We instead hypothesize that the effects we observed result either from impaired wide-field visual motion processing in the optic lobes or during the conversion of visual motion cues to motor commands in the central complex. These hypotheses remain to be tested in future studies.

The doses of imidacloprid used in this study were above field-realistic concentrations in nectar [e.g., 0.5-15 ppb (18, 19)], however they were in the range of nectar concentrations of sulfoxaflor [50-900 ppb (17)]. While the insecticides were sublethal over the exposure period, the resulting effects on visually-guided behavior were dramatic. Even a small impairment of wide-field motion detection could have enormous consequences for flight stabilization. Although the processing of rotational optic flow is not the same as translational optic flow, it is possible that exposure to pesticides could affect visual odometry by impairing wide-field motion processing. Future work should further quantify the effects of field-realistic concentrations on flight behavior to assess whether the effects we observed translate to an impairment of the visual odometer.

## Data availability statement

The datasets presented in this study can be found in online repositories. The names of the repository/repositories and accession number(s) can be found below: https://doi.org/10.5061/dryad.ht76hdrh4.

## Author contributions

RP designed and conceptualized experiments. RP collected and analyzed behavioural and RT qPCR data. CF performed TUNEL assay, confocal microscopy, and image analysis. RP, CF, and JG wrote the manuscript. All authors contributed to the article and approved the submitted version.

## Funding

This study was funded by the 2019 Grass Fellowship in Neuroscience (RP and CF). All experiments were performed at the Marine Biological Laboratory (Woods Hole, MA) with the exception of confocal microscopy, which was performed at the Institute of Neuronal Cell Biology (TUM, Munich, Germany).

## Acknowledgments

We thank the 2019 Grass Foundation Trustees, Director (Melissa J. Coleman), Assistant Director (Christophe Dupre), and the other Fellows and wider MBL community for valuable discussions about this project. We are deeply grateful for the involvement of the Promega ambassadors at the Marine Biological Laboratory, who supplied reagents, equipment, and their time to assist with the RT qPCR optimization. We thank Josh Leveque at Falmouth Academy for access to the honeybees, and Thomas Misgeld (TUM, Munich) for access to a FV1000 confocal microscope.

## Conflict of interest

The authors declare that the research was conducted in the absence of any commercial or financial relationships that could be construed as a potential conflict of interest.

## Publisher’s note

All claims expressed in this article are solely those of the authors and do not necessarily represent those of their affiliated organizations, or those of the publisher, the editors and the reviewers. Any product that may be evaluated in this article, or claim that may be made by its manufacturer, is not guaranteed or endorsed by the publisher.

## Supplementary material

SUPPLEMENTARY FIGURE S1 RT qPCR amplification. Amplification **(A)**, standard curve **(B)**, and melt curves **(C)** for a control sample containing RNA extracted from 5 honeybee brains.

SUPPLEMENTARY FIGURE S2 Optic lobe staining. Optic lobe sections with TUNEL staining (and Hoechst for nuclei) with out (lefthand images) or with DNase I treatment as negative and positive controls, respectively. Scale bar is 100 μm.

SUPPLEMENTARY FIGURE S3 Volume consumed and survival of bees over 5 days. **(A)** The average volume consumed per bee per day did not vary by treatment (Kruskal-Wallis, $(\chi_{3}^{2}$ = 2.21, p = 0.53, n=26 cages per treatment). **(B)** Survival of bees over 5 days did not vary by treatment (Cox proportional hazard, likelihood ratio test=1.91, df = 3, p = 0.59. n=130 bees per treatment.

SUPPLEMENTARY FIGURE S4 Within-animal variation in accumulated rotation between stimulus presentations. Each leftward and rightward stimulus was presented 4 times (in sequence left, right, left, etc.). There was no significant difference between treatments in the standard deviation of the accumulated rotation of individual bees (LME, F_3,98_ = 2.01, p = 0.118), while there were differences across stimuli (LME, F_10,1112_ = 14.0, p < 0.0001).

SUPPLEMENTARY FIGURE S5 Consensus clustering validation results. **(A–D)** Optimal number of clusters (k) was determined using the Monte Carlo reference-based consensus clustering (M3C) package. The cumulative distribution function (CDF, A), relative cluster stability index (RCSI, B), Monte Carlo p-value **(C)** and Entropy **(D)** demonstrate that k=3 is the optimal number of clusters, and that these clusters fit the data significantly better than k=1 clusters. **(E)** Two-dimensional visualization of k-means clustering with 3 clusters. **(F)** Consensus matrices with 2, 3 or 4 clusters. Highest cluster consensus is found with three clusters, visualized with the consensus matrices which compare clustering runs across iterations. Dark blue areas represent high cluster consensus between runs, while white areas represent samples that never cluster together.

SUPPLEMENTARY VIDEO S1 Optomotor behaviour tracking using FicTrac to extract the accumulated rotation. The video panels on the left side show the output from FicTrac, including the input video of the bee walking on the air supported ball, the tracking of the rotation of the ball, and the extrapolated flat walking path. The right panel shows the corresponding accumulated rotation, with upwards motion representing the rightwards direction, and downwards is leftwards. Asterisks appear at the time the stimulus instantaneously switches direction. The visual stimulus grating has a spatial frequency of 0.0625 cpd and a temporal frequency of 16 Hz. The bee is from the control group.
