# Supplementary figures and images for: Chronic exposure to insecticides impairs honeybee optomotor behaviour
Source: Front Insect Sci. 2022 Aug 17;2:936826. doi: 10.3389/finsc.2022.936826 (PMC10926483; doi:10.3389/finsc.2022.936826)

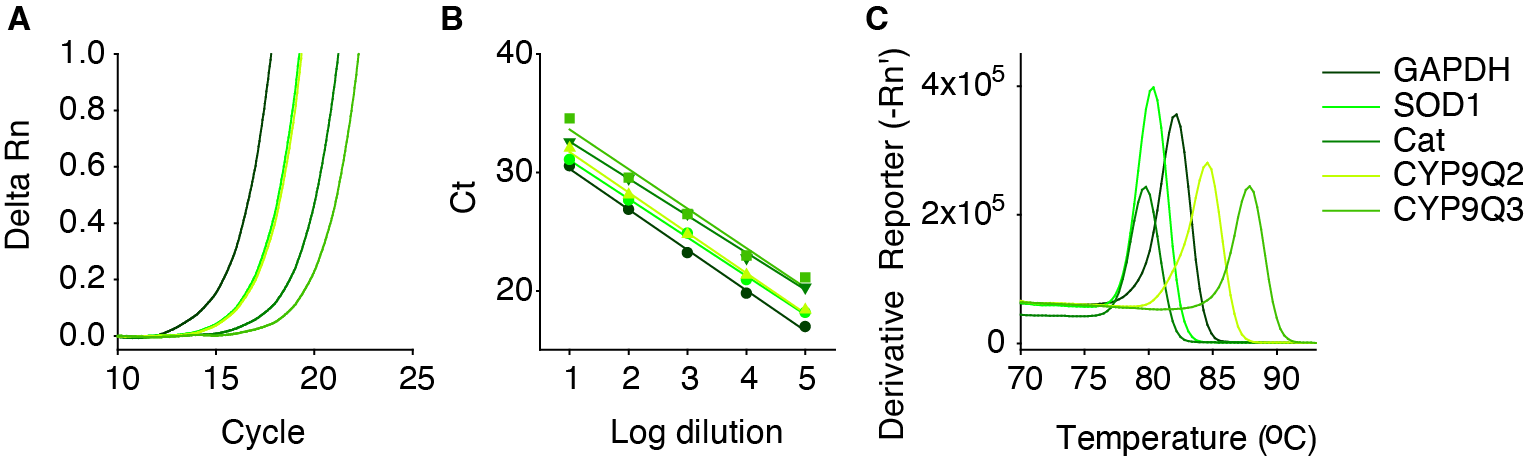

Supplement: SUPPLEMENTARY FIGURE S1 — RT qPCR amplification. Amplification (A), standard curve (B), and melt curves (C) for a control sample containing RNA extracted from 5 honeybee brains. [file Image_1.tif]

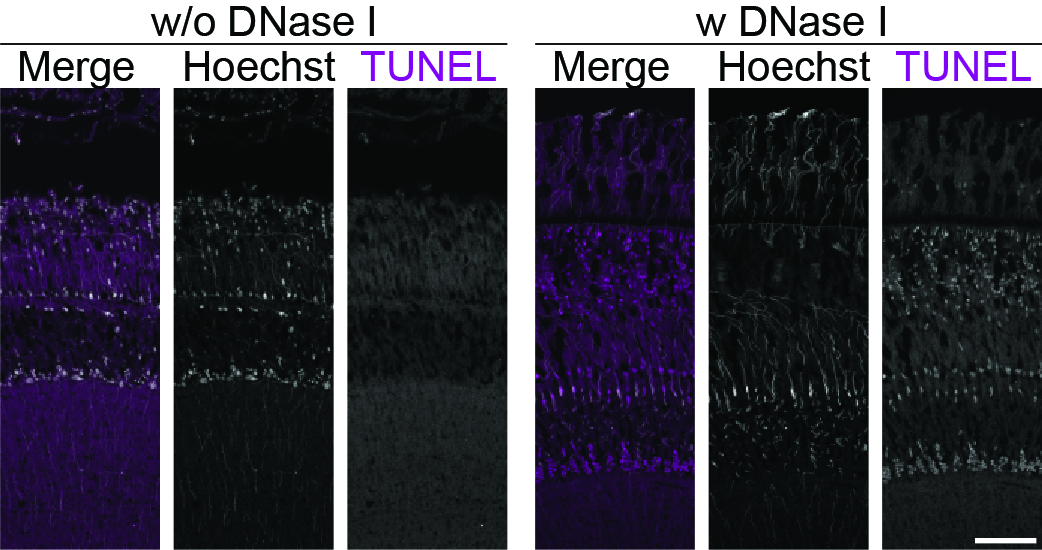

Supplement: SUPPLEMENTARY FIGURE S2 — Optic lobe staining. Optic lobe sections with TUNEL staining (and Hoechst for nuclei) with out (lefthand images) or with DNase I treatment as negative and positive controls, respectively. Scale bar is 100 μm. [file Image_2.tif]

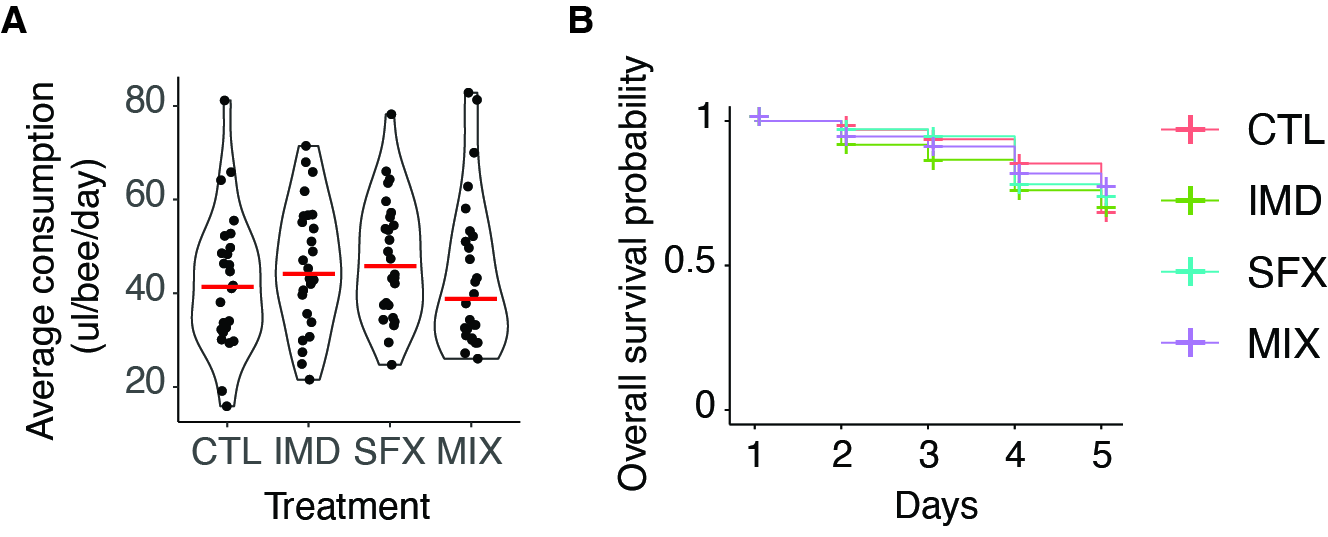

Supplement: SUPPLEMENTARY FIGURE S3 — Volume consumed and survival of bees over 5 days. (A) The average volume consumed per bee per day did not vary by treatment (Kruskal-Wallis, (χ32 = 2.21, p = 0.53, n=26 cages per treatment). (B) Survival of bees over 5 days did not vary by treatment (Cox proportional hazard, likelihood ratio test=1.91, df = 3, p = 0.59. n=130 bees per treatment. [file Image_3.tif]

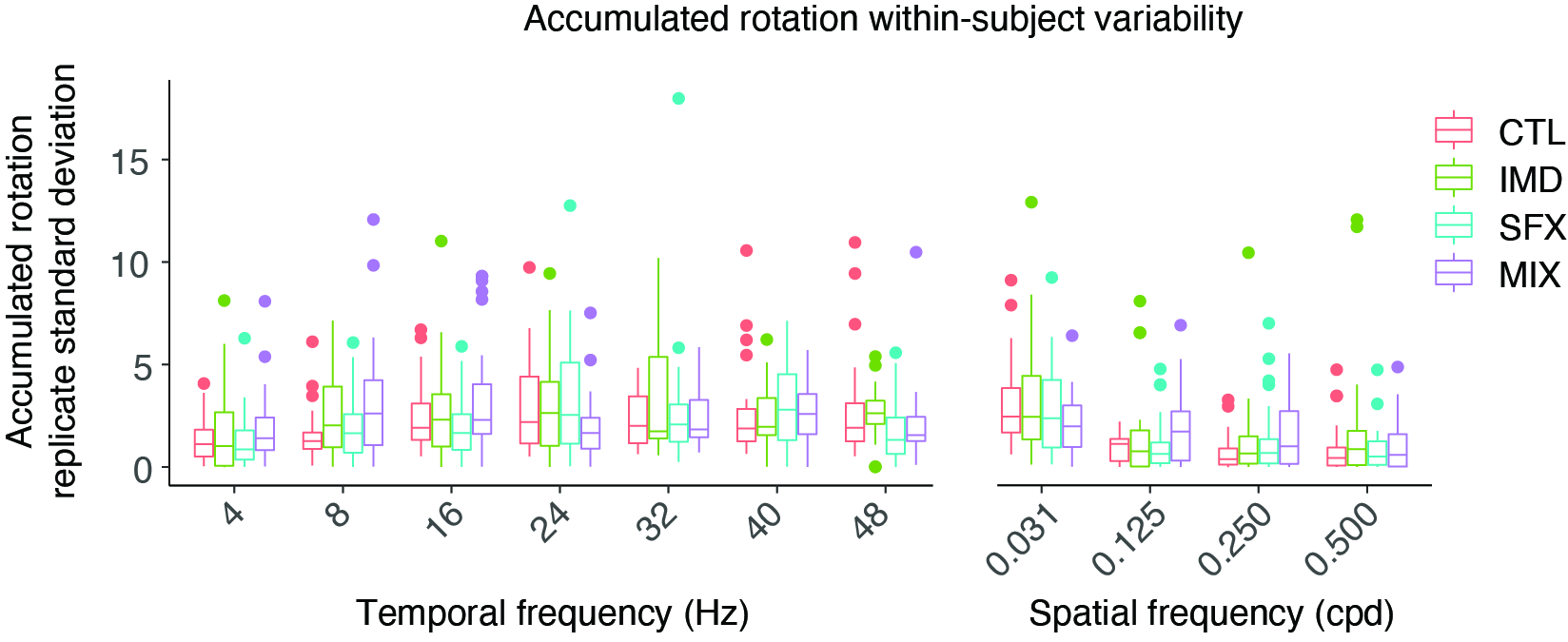

Supplement: SUPPLEMENTARY FIGURE S4 — Within-animal variation in accumulated rotation between stimulus presentations. Each leftward and rightward stimulus was presented 4 times (in sequence left, right, left, etc.). There was no significant difference between treatments in the standard deviation of the accumulated rotation of individual bees (LME, F3,98 = 2.01, p = 0.118), while there were differences across stimuli (LME, F10,1112 = 14.0, p < 0.0001). [file Image_4.tif]

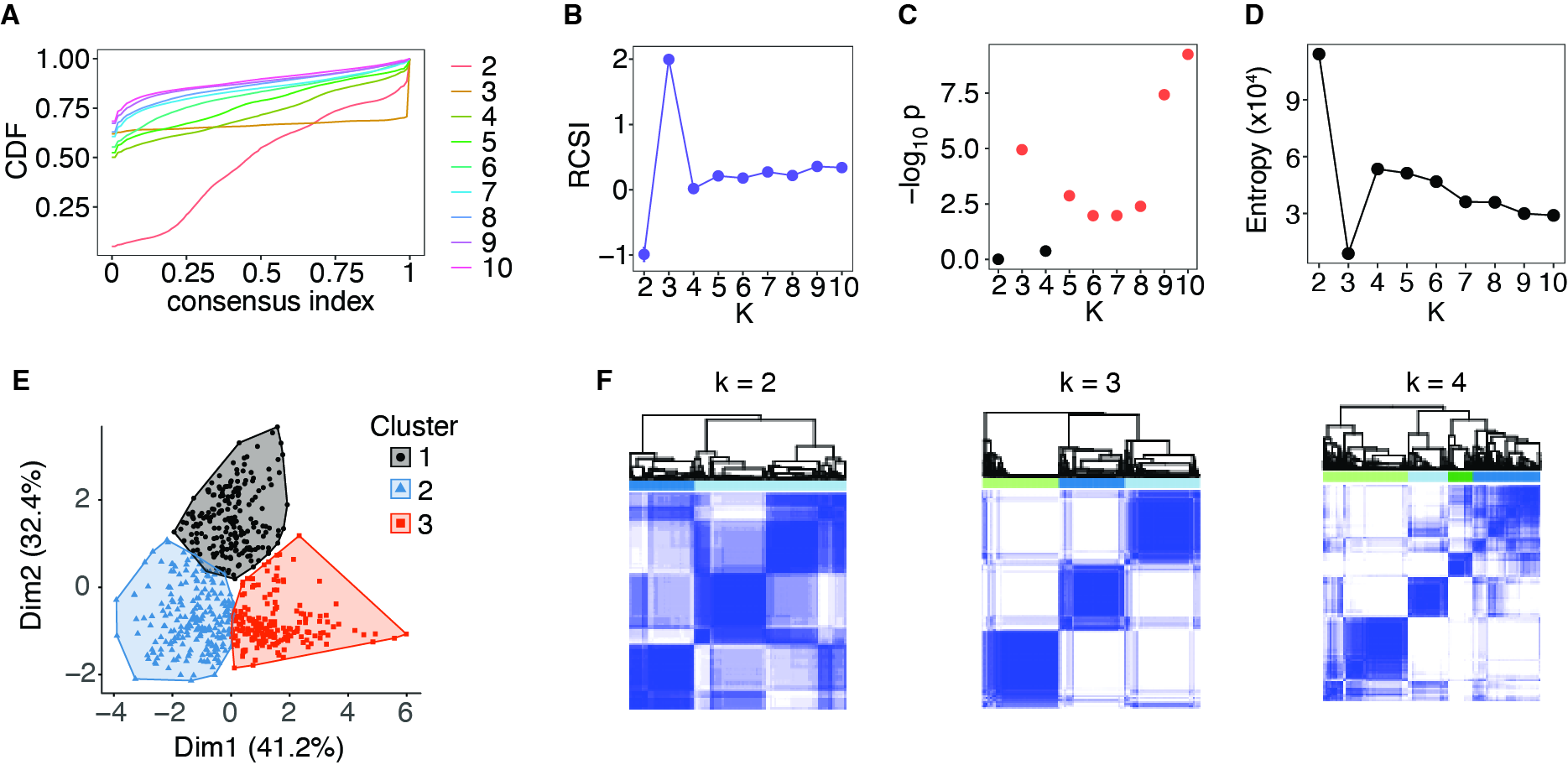

Supplement: SUPPLEMENTARY FIGURE S5 — Consensus clustering validation results. (A–D) Optimal number of clusters (k) was determined using the Monte Carlo reference-based consensus clustering (M3C) package. The cumulative distribution function (CDF, A), relative cluster stability index (RCSI, B), Monte Carlo p-value (C) and Entropy (D) demonstrate that k=3 is the optimal number of clusters, and that these clusters fit the data significantly better than k=1 clusters. (E) Two-dimensional visualization of k-means clustering with 3 clusters. (F) Consensus matrices with 2, 3 or 4 clusters. Highest cluster consensus is found with three clusters, visualized with the consensus matrices which compare clustering runs across iterations. Dark blue areas represent high cluster consensus between runs, while white areas represent samples that never cluster together. [file Image_5.tif]
