# Supplementary material for: Chronic exposure to insecticides impairs honeybee optomotor behaviour
Source: Front Insect Sci. 2022 Aug 17;2:936826. doi: 10.3389/finsc.2022.936826 (PMC10926483; doi:10.3389/finsc.2022.936826)
Supplement: Supplementary file 7 [file DataSheet_1.pdf]

**Table S1. Spatiotemporal properties of optic flow stimuli and number of bees tested of total sample size (CTL n=22, IMD n=25, SFX n=28, and MIX n=25**

| Spatial freq (cpd) | Temporal freq (Hz) | Velocity (deg/s) | CTL n= | IMD n= | SFX n= | MIX n= |
|---|---|---|---|---|---|---|
| 0.0625 | 4 | 64 | 13 | 14 | 15 | 12 |
| 0.0625 | 8 | 128 | 14 | 13 | 16 | 12 |
| 0.0625 | 16 | 256 | 22 | 25 | 28 | 25 |
| 0.0625 | 24 | 384 | 11 | 11 | 16 | 11 |
| 0.0625 | 32 | 512 | 11 | 12 | 13 | 12 |
| 0.0625 | 40 | 640 | 12 | 11 | 14 | 10 |
| 0.0625 | 48 | 768 | 13 | 9 | 12 | 10 |
| 0.03125 | 16 | 512 | 14 | 13 | 16 | 13 |
| 0.125 | 16 | 128 | 13 | 14 | 15 | 10 |
| 0.25 | 16 | 64 | 11 | 12 | 13 | 12 |
| 0.5 | 16 | 32 | 13 | 14 | 15 | 10 |

**Table S2. sequences of oligonucleotide primers for 1-step RT-qPCR used in this study.**

| Primer | Sequency (forward / reverse) | Amplicon length | Accession # | Source |
|---|---|---|---|---|
| GAPDH | 5'-ACCTTCTGCAAAATTATGGCGA-3'<br>5'-CACCTTTGCCAAGTCTAACTGTTAAG -3' | 188 bp | XM_393605 | Collins et al 2004 |
| SOD1 | 5' -AGCACTTGTCGTTCCGTGT A -3'<br>5' -CGGAATTGGTACTCTCCGGT T -3' | 117 bp | NM_001178027 | This study |
| AmCAT | 5' -GGCGGCTGAATTAAGTGCT A -3'<br>5' -TTGCGTTGTGTTGGAGTCA T -3' | 123 bp | NM_001178069 | Reim et al 2013 |
| CYP9Q2 | 5' -CGACACCGTCTCTTCCCAA A- 3'<br>5' -GACAATTGGCCGTTGTTGC T- 3' | 120 bp | XM_392000 | This study |
| CYP9Q3 | 5' -GATGTGCGTCGAGAGTTTC C -3'<br>5' -CTGTCCGGGTCGAATTTGT C -3' | 150 bp | XM_006562300 | Collins et al 2004 |
